# Association between Dietary Habits and Type 2 Diabetes Mellitus in Yangon, Myanmar: A Case–Control Study

**DOI:** 10.3390/ijerph182111056

**Published:** 2021-10-21

**Authors:** Satomi Ueno, Myo Nyein Aung, Motoyuki Yuasa, Ahmad Ishtiaq, Ei Thinzar Khin, Tint Swe Latt, Saiyud Moolphate, Setsuko Sato, Takeshi Tanigawa

**Affiliations:** 1 Department of Public Health, Juntendo University School of Medicine, Juntendo University, Tokyo 113-8421, Japan; sa-ueno@juntendo.ac.jp (S.U.); moyuasa@juntendo.ac.jp (M.Y.); ahmad@juntendo.ac.jp (A.I.); k-ei@juntendo.ac.jp (E.T.K.); st-sato@juntendo.ac.jp (S.S.); tataniga@juntendo.ac.jp (T.T.); 2 Department of Nursing, Seisen Jogakuin College, Nagano 380-0921, Japan; 3 Juntendo Advanced Research Institute for Health Sciences, Juntendo University, 2-1-1 Hongo, Bunkyo-ku, Tokyo 113-8421, Japan; 4 Department of Global Health Research, Juntendo University Graduate School of Medicine, Tokyo 113-8421, Japan; 5 Faculty of International Liberal Arts, Juntendo University, Tokyo 113-8421, Japan; 6 Myanmar Diabetes Association (MMDA), Yangon 11211, Myanmar; proftsl@gmail.com; 7 Department of Public Health, Faculty of Science and Technology, Chiang Mai Rajabhat University, Chiang Mai 50300, Thailand; saiyudmoolphate@gmail.com

**Keywords:** type 2 diabetes mellitus, non-communicable diseases, global health, nutrition, seasoning, lifestyle, Myanmar

## Abstract

In Myanmar, the escalating prevalence of type 2 diabetes mellitus (T2DM) and impaired glucose tolerance among adults was recently reported, with the highest prevalence in the Yangon Region. The aim of the present study was to identify the risk factors in dietary habits and their relationship with T2DM in urban Myanmar residents. We conducted a case–control study recruiting 300 individuals aged 25–74 years living in the Yangon Region, consisting of 150 newly diagnosed cases attending a diabetes clinic, and 150 controls, who were community residents and free of diabetes. The case group had a significantly higher consumption of noodles, fish, beans, fermented food and pickles, dried food, topping seasonings, and non-dairy milk products than the control group, whereas they had a lower vegetable intake (more than three servings/day) and fruit intake (more than three servings/day) than the control group. Furthermore, the case group exhibited a higher frequency of some dietary behaviors than the control group, such as (1) having meals with family, (2) skipping breakfast, and (3) eating out. The final model showed that topping seasonings (adjusted odds ratio (aOR) 11.23, 95% confidence interval (CI) 3.08–40.90), more than three servings/day of vegetable intake (aOR 0.18, 95% CI 0.05–0.67), and having meals with family (aOR 2.23, 95% CI 1.05–4.71) were associated with diabetes. The study suggests that Myanmar’s characteristic dietary culture of topping their meals with salty seasonings and sauces and eating multiple dishes together as a family are risk factors associated with T2DM. Our findings may contribute recommendations and opportunities for the primary prevention of T2DM in urban Myanmar.

## 1. Introduction

Diabetes mellitus is a common chronic disease and a primary global health concern. The worldwide increase in diabetes mellitus has been driven by global aging, economic growth, rapid urbanization, and nutritional trends in different income level countries [1]. A total of 463 million people (9.3% of adults 20–79 years) are living with diabetes worldwide; this number is expected to increase to 578 million (10.2%) by 2030 and 700 million (10.9%) by 2045 [2,3]. The incidence of diabetes mellitus has been rising more rapidly in low- and middle-income countries than in high-income countries, and 80% of the diabetes mellitus cases worldwide live in less developed countries and areas [4,5]. Between 2010 and 2030, the number of adults with diabetes has been projected to increase by 20% in developed countries and by 69% in developing countries [6]. 

Myanmar is the largest country in the region of mainland Southeast Asia. Recent studies have reported a high prevalence of type 2 diabetes (T2DM) among adults in the Yangon Region, which is the largest city in Myanmar [7,8]. In 2014, the national prevalence of T2DM in the adult population aged between 25 and 64 years in Myanmar was 10.5%, while impaired glucose tolerance was nearly two times that of diabetes mellitus (19.7%) [9]. Against this background, the Ministry of Health and Sports (MoHS) formulated the National Strategic Plan for Prevention and Control of Non-communicable Diseases (NCDs), a measure designed to work more effectively on reducing NCDs [10]. The plan has strengthened the medical service system and intervention at the community level to deliver a Basic Essential Package of Health Services to all citizens. However, the specific strategies still have gaps in facilitating lifestyle-related interventions to reduce exposure to key risk factors, including unhealthy diet and physical inactivity. The current study aims to fill this gap.

T2DM develops in association with insufficient insulin action related to decreased insulin secretion or insulin dysfunction through insulin resistance, which involves multiple genetic factors. Insulin resistance is linked to lifestyle habits such as overeating, lack of exercise, and consequent obesity [11,12]. A significant cause of T2DM is an energy-dense Western diet combined with a sedentary lifestyle [13,14]. Based on this evidence, numerous studies have identified the risk factors for T2DM worldwide. In particular, there is a wide range of studies reporting on dietary habits, such as the type of food, food intake, nutrients, dietary patterns, and dietary behavior. Such evidence is still limited in Myanmar, where the culinary tradition is comprised of common Asian-style rice-based meals with family and multiple unique dishes of oily foods, while instant noodles and fried snacks are popular street foods.

The similarities in previous studies on the association between dietary patterns and T2DM are summarized as follows. Healthy dietary patterns characterized by high consumption of foods such as fruits, vegetables, fish, poultry, and whole grains have been associated with a reduced risk of T2DM, whereas unhealthy dietary patterns characterized by a high intake of foods such as processed and red meats, fried products, sweets and desserts, and refined grains have been connected to an increased risk of T2DM [15,16,17,18].

There is increasing evidence that dietary behaviors such as skipping breakfast, speed eating, eating out, and having meals with family are directly associated with obesity and other chronic diseases, including cardiovascular disease and T2DM [19,20,21,22]. As dietary behavior is based on individual lifestyles, such as food selection, frequency, and intake, understanding the mechanism can lead to recommendations for the prevention and management of NCDs, including T2DM.

Previous studies in Myanmar have provided several reports on dietary habits and NCDs, including T2DM. A study focusing on risk factors for NCDs in the Yangon Region showed metabolic risk factors, as well as moderate or high 10-year coronary heart diseases risk, were more common among urban residents, and behavioral risk factor levels tended to be higher among rural areas. In particular, it was found that rural areas consume more alcohol and consume fewer fruits and vegetables than urban areas [23]. Furthermore, in a report investigating the association between fruit and vegetable intake and risk factors for NCDs, a high intake of fruit and vegetables was associated with lower odds of hypertriglyceridemia among men and women [24]. A study comparing dietary intake, dietary patterns, and an abnormal blood glucose status of middle-aged adults in urban and suburban areas of Mandalay found that dietary intake was significantly different between urban and suburban areas. However, the association between food patterns and abnormal glycemic status was not definitively reported [25].

Contrary to the potential importance of dietary nutritional behavior for the increase in T2DM in Myanmar, dietary habits have been only marginally evaluated. Therefore, we conducted a case–control study to identify the specific dietary habits of the adult residents in Yangon. The aim of the present study was to identify the risk factors in dietary habits and their relationship with T2DM in urban Myanmar residents. 

## 2. Materials and Methods

### 2.1. Study Design and Participant Recruitment

This was an observational study applying a case–control study design. It recruited participants who met the eligibility criteria with a ratio of 1:1 to investigate the relationship between dietary habits and T2DM in urban Myanmar. Participants in this study were recruited from December 2018 to February 2019 until the target sample size was reached. A total of 150 case participants and 150 control participants were enrolled from the Yangon Region.

#### 2.1.1. Eligibility Criteria for the Case Participants

**Figure 1:**
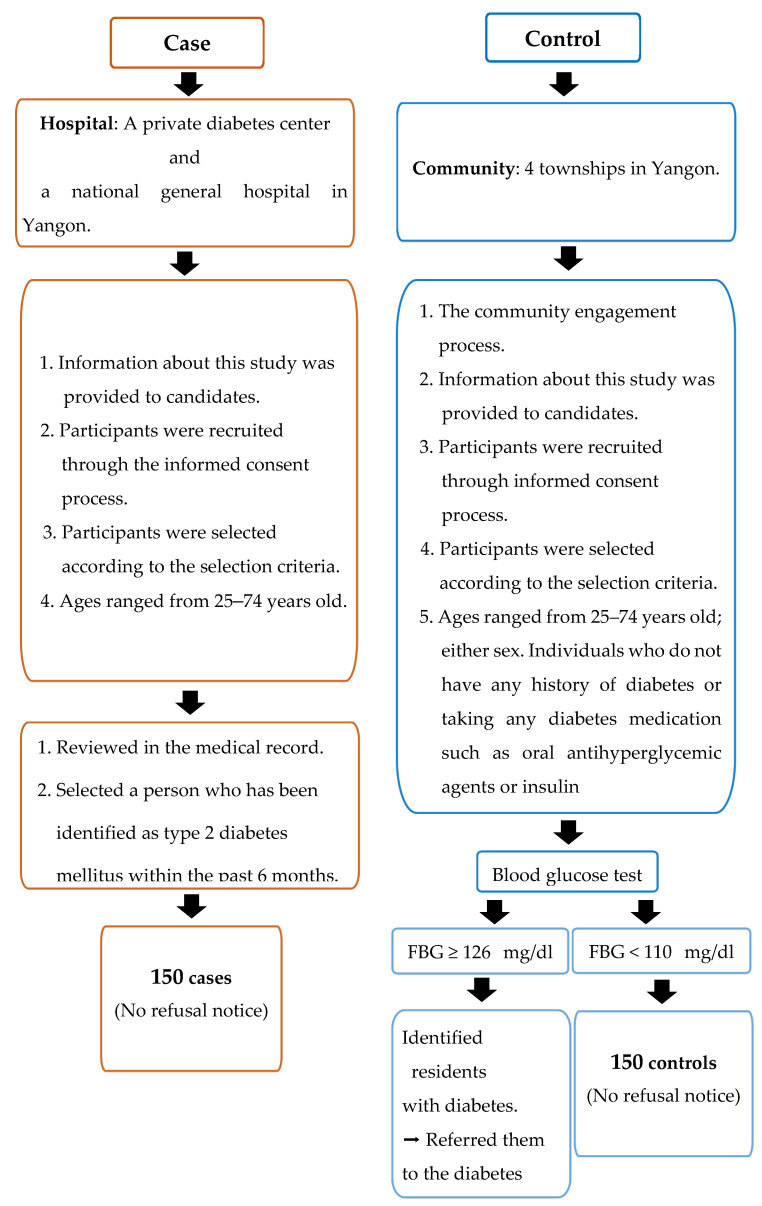
Criteria to select the study population.

Case definition: cases met eligibility criteria (Figure 1) and were diagnosed within six months before data collection. Newly diagnosed diabetes patients attending diabetes clinics were identified by physicians and cross-checked with their medical records before being invited to interviews. Study sites for recruiting cases were a private diabetes center and a national general hospital in Yangon where T2DM diagnosis adhered to the World Health Organization (WHO) diagnosis criteria of diabetes [26].

The eligibility criteria for the case participants were: (1) patients who were newly diagnosed with T2DM within the six months prior to data collection (after June 2018); (2) adults aged 25 to 74 years; (3) either sex; (4) diagnosed by fasting plasma glucose ≥ 126 mg/dL and/or random blood glucose (RBG) ≥ 200 mg/dL, with or without the osmotic symptoms of diabetes (polyuria, polydipsia, thirst, bodyweight loss); and (5) all races and religions, and residing in Yangon [27] (see Figure 1).

#### 2.1.2. Eligibility Criteria for the Control Participants

Control definition: controls were community controls randomly recruited from four townships in Yangon. Controls were defined as community residents who are eligible to participate in the study according to the criteria and free of diabetes.

The eligibility criteria for the control participants were: (1) adults aged 25 to 74 years; (2) either sex; (3) individuals who do not have a history of diabetes or taking any diabetes medication, such as oral antihyperglycemic agents or insulin; (4) fasting blood glucose < 110 mg/dL and random blood glucose (RBG) < 140 mg/dL); and (5) all races and religions, and residing in Yangon [27] (see Figure 1). 

#### 2.1.3. Sample Size

The sample size was calculated using the following formula [28]:

(1) $$n=(\frac{r+1}{r})\frac{(\bar{p})(1-\bar{p}){\left(Z_{\beta }+Z_{\alpha /2}\right)}^{2}}{{\left(p_{1}-p_{2}\right)}^{2}}$$

(2) $$P_{1}=0.76\;P_{2}=0.86\;\bar{P}=0.81$$

To compare the dietary habits of the case and control groups, the sample size of this study was estimated by reference to the 2014 results for Myanmar on the WHO STEPwise Approach to NCD Risk Factor Surveillance (STEPS) [29]. In that survey, 86.6% of respondents consumed an average of fewer than five servings of fruits and vegetables a day. Therefore, this rate was assumed to be the rate of exposure to risk factors in the case group (P_1_). Assuming that people without diabetes had a high rate of vegetable intake, the control group rate was set at 76%. As a result of calculating the power used as 80% and the α error as 5% on both sides, the number of samples required to obtain the difference between the case group and the control group was 242. Therefore, 300 samples were sufficient to confirm statistically significant differences between the 80% probability and the 95% confidence intervals. Moreover, the number of samples was able to compensate for the predicted number of non-responders, which was approximately 10%.

### 2.2. Measurements

#### 2.2.1. Definition of Dietary Habits

Dietary habits are habitual decisions an individual or culture makes when choosing what foods to eat and how to eat them [30]. Dietary habits and choices play a significant role in human health [31]. Therefore, in this study, we defined dietary habits as actual food intake and dietary behavior.

#### 2.2.2. Dietary Habits Assessment

Information on dietary habits was collected using the Food Frequency Questionnaire (FFQ), which estimates the frequency of daily food intake over a period. It has been widely used internationally [32].

The food items were selected based on the Association of Southeast Asian Nations (ASEAN) food composition table [33] and those foods that are commonly eaten locally, as follows: rice, bread, noodles, meat, processed meat, fish, seafood, egg, beans, nuts, dairy milk products, non-dairy milk products, deep-fried food, stir-fried food, oil, seasonings, dried food, fermented food and pickles, sweet food, soft drink, fresh fruit juice, coffee or tea, vegetables, and fruit. Participants were asked for portion sizes of vegetables, fruits, rice, bread, soft drink, fresh fruit juice, and coffee or tea. Dietary behavior included the behavioral patterns that are associated with T2DM based on previous studies: having meals with family [22]; skipping breakfast [19]; eating out [21]; eating prepared foods [34]; having snacks [35]; removing visible fat [36]; drinking alcohol [37]; using supplements [38]; using traditional medicine [39]. All participants were asked about the frequency of their dietary habits over the past week: never or very rarely; 1–2 times/week; 3–4 times/week; 5–6 times/week; or every day.

#### 2.2.3. Other Assessments

For those diagnosed with fasting plasma glucose in the case group, the date of diagnosis of T2DM and the blood sampling result were confirmed from medical records by collaborators (two physicians). Participants identified as having T2DM within the last six months were selected. Fasting plasma glucose in the control group was examined by collaborators (two physicians) using blood samples from participants’ capillaries. Blood samples were taken on an empty stomach after fasting for at least 8 h. Persons with fasting plasma glucose < 110 mg/dL were selected. A single investigator took anthropometric measurements using the WHO STEPS protocol [40]. Height was measured in centimeters with participants standing without shoes (0.5 cm accuracy). Weight was measured in kilograms with participants in light clothing (0.1 kg accuracy) using a digital weight scale (OMRON Body Composition Analyzer HBF-375, Japan). Waist circumference and hip circumference were measured using non-elastic plastic tape (TAKACHIHO Medical Co., Ltd., Tokyo, M12DXB, Japan). Body mass index (BMI) was calculated by dividing weight (kg) by height squared (m). The waist-to-hip ratio (WHR) was calculated by dividing the waist circumference by the hip circumference. Blood pressure was measured using the OMRON Digital Automatic Blood Pressure Monitor (HEM-907XL, Japan) after resting for 10 minutes in a sitting position. A trained physician performed all measurements using standardized procedures. Information collected on the demographic characteristics included: age (continuous in years); gender (male, female); marital status (never married/single, currently married, separated, divorced, widowed); education status (no formal schooling, less than primary school, primary school completed, secondary school completed, high school completed, college/university completed, postgraduate degree completed); employment status (government employee, self-employed, daily wager, education or livestock worker, factory or assembly worker, professional worker (e.g., accountant, doctor, academic), skilled worker (e.g., carpenter, hairdresser, computer worker), full-time student, household worker, retired, employed (able to work), unemployed (unable to work, others); personal monthly income (less than average, middle, high, very high); and family history of diabetes (yes, no). We also collected information on behavioral habits such as: cigarette smoking (never, quit, still smoking); alcohol drinking (never, quit, still drinking); and physical activity (vigorous, moderate, walking, sedentary). The International Physical Activity Questionnaire-Short Form (IPAQ-S) [41] was used for measuring physical activity. Measured time was calculated as minutes/week for vigorous physical activity, moderate physical activity, and low physical activity, and minutes/day for sitting (sedentary).

### 2.3. Data Collection

According to the WHO guidelines, the survey instrument and consent cover letter were translated from English to Burmese [42]. Prior to this survey, a pretest using the same questionnaire was conducted on 30 Myanmar nationals who were residing in Tokyo to confirm the accuracy of the translation of the questionnaire into Myanmar. Based on the results, the revised questionnaire was used in this study in Myanmar. The questionnaire survey was conducted on all participants using a structured questionnaire that employed the self-administered questionnaire method. Research assistants (two physicians) who were trained in this study responded to requests for explanations of the questionnaire survey and questions from participants at any time. The questionnaire consisted of five sections: demographic information (demographic characteristics information, social-demographic information); medical information (medical history information, diabetes medication history); behavioral measurements (tobacco and alcohol use, physical activity, dietary habit); physical measurements (height, weight, waist circumference, hip circumference, blood pressure); and biochemical measurements (blood glucose).

### 2.4. Statistical Analysis

Categorical variables were expressed as numerals and percentages and were compared with the chi-squared test. Continuous variables were expressed as mean with standard deviation (SD) and were compared using a Student’s t-test or a Mann–Whitney U test for comparisons in each group. Food intake frequency and dietary behavior were classified into dichotomous categorical variables of high frequency and low frequency: high frequency (daily); low frequency (totally or very rarely, 1–2 times/week, 3–4 times/week, 5–6 times/week); high-frequency intake of vegetables and fruit (≥3 servings/day); and low-frequency intake of vegetables and fruit (<3 servings/day). We calculated the odds ratios (OR) and 95% confidence interval (CI) using univariate logistic regression to identify factors associated with dietary habits and T2DM. The outcome variable was diabetes status as binary data. Identified confounders were controlled in multivariate analysis, using multiple logistic regression that reported adjusted odds ratio (aOR) and 95%CI for each specific dietary habit exposure variable. The selection of covariates for multivariate logistic regression analysis was based on variables that showed a *p*-value < 0.2 in univariate analysis and previous studies. In the first model for the food intake, we adjusted for age and gender. In the second model, marital status, education status, family history of diabetes, current alcohol drinkers, physical activity (vigorous + moderate + walking activity ≥ 420 h/week, vigorous + moderate + walking activity < 420 h/week), and BMI (≥25 to 25<) were added to model 1 for adjustment. In the final, fully adjusted model, we additionally adjusted for having meals with family (never–every day) to model 2. In the first model for dietary behavior, we adjusted for age and gender. In model 2, marital status, education status, family history of diabetes, current alcohol drinkers, physical activity, and BMI were added to model 1 for adjustment. In the final, fully adjusted model, we additionally adjusted model 2 for seasonings (never–every day), vegetables (≥3 servings/day to <3 servings/day), and fruit (≥3 servings/day to <3 serving/day). 

Model fitness was checked using the Hosmer–Lemeshow goodness-of-fit test. A *p*-value < 0.05 was considered to indicate associated factors. Multiple logistic regression and all the analyses were performed using STATA version 16 IC (Stata Corporation, College Station, TX, USA).

### 2.5. Ethical Approval

This study was conducted in accordance with the Declaration of Helsinki principles. The protocol for the study was approved by the Juntendo University Research Ethics Committee (approval number 2017141, 18 January 2018) and the Myanmar Ethics Review Board (approval number PLRID-00625_V1, 2 July 2019). Written informed consent was obtained from all participants.

## 3. Results

We analyzed 150 newly diagnosed T2DM patients and 150 community residents without T2DM. Table 1 shows the general characteristics of the case and the control groups and their associations with other covariates. The number of diabetic females (68.7%) was greater than males (31.3%). The mean age was 55.1 ± 10.9 years in the case group and 43.3 ± 14.8 years in the control group. The case group had a significantly lower level of education, employment, and personal monthly income compared to the control group. The case group had a more secure marital status and a more significant family history of diabetes compared to the control group. There was a significant difference in BMI, WHR, and high blood pressure between the case group and the control group. Regarding physical activity, the case group spent more time walking or in moderate or vigorous physical activity than the control group. Of the diabetes participants, 86% of those in the case group were taking medicine at the time of data collection, whereas no one in the control group was on medication.

**Figure 2:**
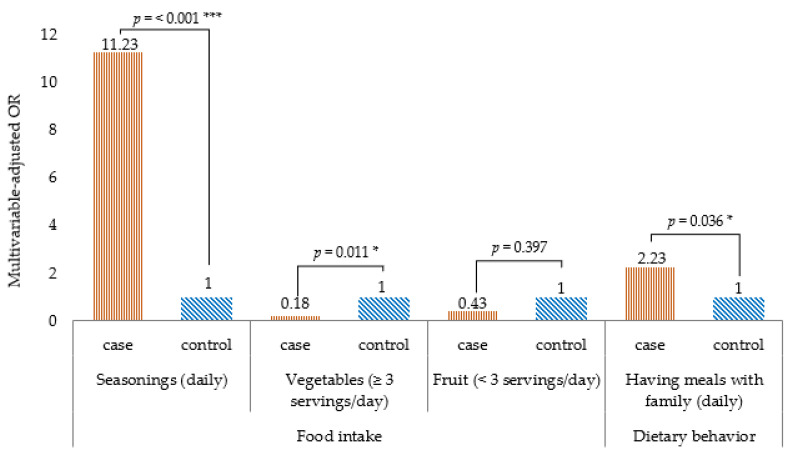
Dietary habits associated with type 2 diabetes mellitus in a case–control study of the urban residents in Yangon, Myanmar, 2019. Note: Multivariable logistic regression analysis; p-value for the comparison between two groups: *** *p* < 0.001, * *p* < 0.05. All *p*-values are two-sided.

Table 2 shows the comparison of food intake frequency by univariate logistic regression analysis, the intake frequency of noodles, fish, beans, fermented foods and pickles, dried foods, and topping seasonings (such as salt, soy sauce, and fish sauce) was higher in the case group than in the control group. Conversely, the intake frequency of non-dairy products, vegetables (three or more servings/day), and fruits (three or more servings/day) was lower in the case group than in the control group. Table 3 shows the relationship between a high food intake frequency and T2DM, the results of multivariate logistic regression analysis. Statistically significant association of variables such as noodles, fish, beans, fermented foods and pickles, dried food, non-dairy milk products, and fruit to the outcome disappeared after adjusting for age, gender, marital status, education status, marital status, family history of diabetes, current alcohol drinkers, physical activity, BMI, and having meals with family. The final model showed that topping seasonings (aOR 11.23, 95% CI 3.08–40.90) was associated with a higher risk of T2DM, whereas more than three servings/day of vegetables (aOR 0.18, 95% CI 0.05–0.67) was associated with lower risk of T2DM (Figure 2).

Table 4 shows the comparison of the frequency of dietary behavior by univariate regression analysis. The frequency of having meals with family, skipping breakfast, and eating out was higher in the case group than in the control group. Table 5 shows the relationship between the high frequency of dietary behavior and T2DM by multivariate logistic regression analysis. The association of variables skipping breakfast and eating out turned out to be non-significant after adjusting for age, gender, marital status, education status, family history of diabetes, current alcohol drinkers, physical activity, BMI, seasonings, vegetables, and fruit. The final model showed that having meals with family (aOR 2.23, 95% CI 1.05–4.71) was associated with T2DM (Figure 2). 

## 4. Discussion

This study aimed to identify the risk factors in dietary habits and their relationship with T2DM in urban Myanmar residents. As a result, a daily intake of seasonings was significantly associated with T2DM, and a vegetable intake of three or more servings daily was inversely associated with T2DM. Regarding dietary behavior, having meals with family was associated with T2DM.

We defined seasonings as salty foods such as salt, soy sauce, fish sauce, and fish paste. According to a study in Lithuania, after adjusting for possible confounders, participants who added salt to prepared meals had about a two-fold higher risk of developing T2DM compared to participants who never added salt to prepared meals [43]. In the first systematic review and meta-analysis assessing the relationship between sodium status and T2DM in adults, patients with T2DM had significantly higher sodium levels than controls. Regarding sodium intake, patients with T2DM had substantially higher levels of sodium intake compared to non-diabetic controls. Furthermore, increased urinary sodium excretion was associated with a higher risk of developing T2DM [44]. A prospective study in Finland reported that the relationship between sodium intake and risk of T2DM based on 24-h urinary sodium excretion data showed that high levels of sodium intake, measured in the highest quartile of 24-h sodium excretion, significantly increased the risk of T2DM [45]. In a recent study by Abdulai et al. on the role of a high dietary salt intake preference and diabetes in a rural population in China, the preference for a higher intake of dietary salt was associated with undiagnosed diabetes but not prevalent diabetes [46]. 

Moreover, other studies have demonstrated that an excessive salt intake may increase the risk of developing T2DM, possibly through a direct effect on insulin resistance and/or by promoting high blood pressure and weight gain [46,47]. The following presumed mechanisms have been proposed for the association between salt intake and T2DM. In general, obesity is associated with overnutrition and an excessive intake of sodium-rich foods [47]. A high salt intake activates the aldose reductase–fructokinase pathway in the liver and hypothalamus, leading to leptin resistance and endogenous fructose production, which affects obesity, insulin resistance, and fatty liver disease [48]. As a result, it may increase the risk of developing T2DM by directly affecting insulin resistance and/or promoting hypertension and weight gain [47,48]. A recent study by the Karolinska Institute for Environmental Medicine in Sweden (2017) found that for every 2.5 g of salt intake, the risk of developing T2DM increased by 65%. This study also revealed that the risk of developing T2DM is increased by 72% in people who consume more salt (7.3 g/day or more) than those who take less salt (less than 5.8 g/day) [47]. 

Nga Pi, a paste made from salted fermented fish or shrimp, is the main ingredient in a traditional food in Myanmar and is used as a condiment or additive in most dishes. It is a versatile food with many uses, such as in soup bases, salads, main dishes, and condiments [49]. Furthermore, Myanmar has a variety of other salty seasonings (pastes and sauces). While our study has not identified the kind of seasoning the participants added, the previous studies mentioned above support our result that Myanmar’s unique dietary habit of adding these salty seasonings to cooked meals may contribute to an increased prevalence of T2DM.

Patients with diabetes are sensitive to salt, and salt intake is a major risk factor for increasing blood pressure. Moreover, hypertension is one of the leading causes of the accelerated progression of nephropathy [50]. Therefore, salt reduction education for patients with diabetes mellitus may lead to the prevention of diabetic nephropathy [51], and it can be necessary to promote it.

Vegetables and fruit are rich in antioxidants, such as polyphenols, carotenoids, and vitamin C, which have been associated with a decreased risk of T2DM [52,53]. A meta-analysis of prospective studies found an association between vegetable and fruit intake and a lower risk of T2DM [52,54]. In addition, there are reports that green leafy vegetables reduce the risk of developing T2DM [55]. On the other hand, another prospective study found no association between vegetable and the risk of T2DM [56]. Other studies showed that the intake of vegetables and fruit combined, vegetables only, and fruit only were not significantly associated with the risk of T2DM [55,57]. Similarly, our results showed that only a daily vegetable intake of three servings or more was inversely associated with the risk of T2DM.

A previous study followed the WHO STEPwise approach to the surveillance of chronic disease risk factors in Yangon [58]. The study reported the behavioral risks of NCDs, such as a high alcohol intake and a low vegetable and fruit intake in rural areas [23]. According to a study comparing the results of the 2009 and 2014 WHO STEPS surveys, the number of individuals with low vegetable/fruit consumption (<5 servings) has declined (4.3% RC), yet a high percentage of individuals were still observed as having <5 servings of vegetables and/or fruit per day (90.5% in 2009 and 86.6% in 2014) [59]. Another study found that those with at least two servings per day of vegetables and fruit had lower odds than others for hypertriglyceridemia among men and women in the Yangon Region and also had lower levels of total cholesterol among women [24]. Hypertriglyceridemia is one of the most common lipid abnormalities encountered in clinical practice. The most common causes of hypertriglyceridemia are obesity and uncontrolled diabetes [60]. Therefore, these results may support our findings that a high vegetable intake is inversely associated with the risk of T2DM among Yangon residents. 

We found that having meals with family was significantly associated with an increased risk of T2DM. Previous studies have emphasized the health benefits of frequently having meals with family to children [61], adolescents [62], adults [63,64], and older adults [22]. The outcomes of these studies include promoting a healthy diet and reducing the risk of overweight and obesity. Meanwhile, Horikawa et al. reported that energy intake was significantly higher in diabetic patients who ate at least once a month with their families than in those who did not [65]. In addition, Jeong et al. showed that triglycerides and fasting blood glucose in older males were likely to decrease as the frequency of having meals with family increases, conversely revealing conflicting results with those of elderly females. Elderly females had a greater total energy as a result of the frequency of having meals with their family; further, it showed an increased intake of nutrients, except for fat [22]. Those findings may support our results. 

However, in our study, the case group had a higher proportion of females, older ages, and married participants compared to the control group. In Asia, including Myanmar, females are generally responsible for most of the household chores. In preparing meals, females repeatedly taste food and take care of the food leftover after eating [22]. Hence, it is undeniable that the difference in social background between the case group and the control group affected our results.

Moreover, the traditional Myanmar dining style is to sit on a mat around the table and share a meal with the family [49], a custom that may have led to excessive dietary intake in participants who frequently ate with their families. 

Therefore, the evidence of a relationship between having meals with family and health outcomes is inconsistent [62,63]. Accordingly, the mechanism that explains the association between the frequency of having meals with family and adult health status is unclear. However, it can be hypothesized that healthier meals served with family members helped to improve the nutritional intake and health quality of the adult participants. Moreover, we found that the education status was significantly lower (<primary school) in the case group than in the control group. This result implies that in order to stop the increase in T2DM in Yangon, it is necessary to promote adequate nutrition education simultaneously, rather than focusing solely on the frequency of having meals with family. In short, it can be assumed that having meals with family and sharing meals can create more meaningful opportunities for family members to share knowledge and information regarding daily food habits, which may lead to behavioral changes to acquire healthy lifestyle habits.

In summary, overnutrition as a result of having meals with family may have induced obesity, a major risk factor for T2DM. Fulkerson et al. suggested that eating with family, friends, or neighbors in a home environment may differ from eating with others in a congregate meal setting or alone [63]. In short, this context may have a larger effect on dietary intake or weight status. However, our research has not yet evaluated the potential factors that are particularly related to having meals with family, such as the amount, types, and sources of foods served. Further studies are required to better understand the complex association between having meals with family and the increased prevalence of T2DM. Notably, diabetes prevention in Myanmar could apply family-based prevention approaches, such as information on how to modify meals with the family to be healthier.

This study has several strengths. First, a case–control study was designed to identify the factors associated with dietary habits and T2DM, with cases selected from a hospital site and controls selected from a community site selected independently of exposure. Second, we were able to adjust for various potential confounders, including age, gender, education status, physical activity, and other health statuses that could disrupt the association between dietary habits and T2DM by multivariate logistic analysis. 

This study had limitations. First, there is a possibility of recall bias in the nature of the case–control study. Participants responded to questions regarding their dietary habits over the previous seven days by applying FFQ to detect their dietary habits. Second, some participants in the case group may have changed their lifestyle behaviors, including dietary and physical activity habits, during six months after the initial consultation as they may have already obtained information on lifestyle. Food habits are relatively quick to adopt. This may have reduced the difference in some food habits between the case and control groups. As is the nature of case–control studies, readers should interpret epidemiological relationships between diabetes and risk factors carefully, with biological plausibility. Finally, there was a large difference in the attributes of the case group and the control group. In Myanmar, it was difficult to freely select the target area due to the restrictions of the political system. Participants in both groups were randomly chosen to minimize the selection bias; however, significant differences in attributes occurred due to characteristic differences between hospitals and communities. 

The present study was the first to identify the factors behind the increased prevalence of T2DM in urban Myanmar by focusing on dietary habits. Therefore, our findings may contribute to the formation of a prevention program based on cultural dietary habits in a community setting as a health promotion intervention for T2DM. We recommend that further longitudinal studies be undertaken to investigate the association between seasoning use and T2DM, and an intervention study targeting healthy family meals in Myanmar, such as cooking classes and early nutritional education. Public health interventions, with either appropriate risk targeting or population-wide interventions, to suppress the rise in diabetes mellitus are required in Myanmar and in many countries that share similar cultures and contexts [10,66].

## 5. Conclusions

We clarified in detail the different dietary habits of diabetes patients and non-diabetes persons in Yangon, Myanmar. The study suggests that Myanmar’s characteristic dietary custom of topping meals with salty seasonings and sauces and eating multiple dishes together as a family are risk factors associated with T2DM. The study results warn against the use of these seasonings and their effect on T2DM, and it promotes vegetable intake and the empowerment of families to cook and eat healthy meals to prevent the escalating prevalence of diabetes. Our findings contribute recommendations and opportunities for the primary prevention of T2DM in urban Myanmar.

## Figures and Tables

**Table 1:** General characteristics of cases and controls.

	Case (*n* = 150)	Control (*n* = 150)	*p*-Value
Gender, *n*(%)			0.017 *
Male	47(31.3)	67(44.7)	
Female	103(68.7)	83(55.3)	
Age, (mean ± SD)	55.1 ± 10.9	43.3±14.8	<0.001 ***
BMI (kg/m^2^), (mean ± SD)	26.8 ± 4.9	25.0±4.6	0.013 *
WHR (male ≥ 0.9, female ≥ 0.85), *n*(%)	114(76.0)	64(42.7)	<0.001 ***
High blood pressure (SBP ≥ 140, DBP ≥ 90), *n*(%)	20(15.5)	9(6.8)	0.024 *
Physical activity (≥420 mins/week), *n*(%)	83(55.3)	60(40.0)	0.008 **
Marital status (currently married), *n*(%)	99(66.0)	72(48.0)	0.002 **
Education status (primary school or lower), *n*(%)	50(33.6)	13(8.7)	<0.001 ***
Employment status (employed), *n*(%)	69(46.3)	109(73.2)	<0.001 ***
Personal monthly income (low: less than average), *n*(%)	61(41.8)	25(17.1)	<0.001 ***
Current smokers, *n*(%)	4(2.7)	18(12.0)	0.002 **
Current alcohol drinkers, *n*(%)	6(4.0)	38(25.7)	<0.001 ***
Taking diabetes medication, *n*(%)	129(86.0)	―	―
Family history of diabetes, *n*(%)	89(59.3)	69(46.0)	0.021 *

Note: Student’s t-test or Mann–Whitney U test was used to compare continuous variables, and chi-squared test was used to compare categorical variables; SD: standard deviation, %: ratio of valid responses; *p*-value for the comparison between two groups: *** *p* < 0.001, ** *p* < 0.01, * *p* < 0.05. All *p*-values are two-sided.

**Table 2:** Comparison of food intake by high frequency between diabetes and non-diabetes participants.

Food Item		Case (*n* = 150)		Control (*n* = 150)		*p*-Value
Rice	*n*(%)	139	(93.9)	138	(92.0)	0.518
Bread		9	(6.1)	4	(2.7)	0.149
Noodles		15	(10.1)	5	(3.36)	0.021 *
Meat		56	(37.3)	42	(28.0)	0.085
Fish		17	(11.3)	4	(2.67)	0.003 **
Sausage		0	(0.0)	0	(0.0)	―
Seafood		0	(0.0)	3	(2.0)	0.082
Beans		13	(8.8)	4	(2.7)	0.023 *
Nuts		4	(2.7)	1	(0.7)	0.176
Stir-fried food		33	(22.2)	28	(18.8)	0.473
Deep-fried food		8	(5.3)	3	(2.0)	0.127
Fermented food and pickles		9	(6.0)	1	(0.7)	0.010 *
Dried food		6	(4.0)	0	(0.0)	0.014 *
Seasonings		144	(96.0)	101	(69.2)	<0.001 ***
Diary milk product		17	(11.4)	13	(8.7)	0.430
Non-diary milk product		5	(3.3)	15	(10.1)	0.020 *
Dessert		8	(5.3)	11	(7.4)	0.468
Soft drink		1	(0.7)	3	(2.0)	0.314
Fresh fruit juice		2	(1.3)	1	(0.7)	0.566
Coffee and/or tea		40	(26.9)	50	(33.3)	0.221
Vegetables (≥3 servings/day)		7	(4.8)	31	(21.2)	<0.001 ***
Fruit (≥3 servings/day)		2	(1.8)	12	(10.0)	<0.01 *

Note: Chi-squared test was used to compare categorical variables; High frequency: eating every day, %: ratio of valid responses; *p*-value for the comparison between two groups: *** *p* < 0.001, ** *p* < 0.01, * *p* < 0.05. All *p*-values are two-sided.

**Table 3:** Dietary habits associated with type 2 diabetes mellitus among the urban residents in Yangon Myanmar, 2019.

		Association of Dietary Habits to T2DM		
Food Intake	OR	aOR	95% CI	*p*-Value
Seasonings				
Crude	10.65		3.57–31.84	<0.001 ***
Model 1		13.46	5.11–35.42	<0.001 ***
Model 2		11.05	3.93–31.10	<0.001 ***
Model 3		11.23	3.08–40.90	<0.001 ***
Vegetables				
Crude	0.16		0.05–0.48	<0.001 ***
Model 1		0.24	0.09–0.59	0.002 **
Model 2		0.21	0.08–0.56	0.002 **
Model 3		0.18	0.05–0.67	0.011 *
Fruit				
Crude	0.39		0.07–2.16	0.280
Model 1		0.21	0.04–1.02	0.053
Model 2		0.15	0.03–0.84	0.031 *
Model 3		0.43	0.06–3.05	0.397

Note: Multivariable logistic regression analysis; Goodness-of-fit test for a logistic regression model 3; Pearson chi-squared test (*p* = 0.465), Hosmer–Lemeshow chi-squared test (*p* = 0.106); Crude: unadjusted; Model 1: adjusted for age, gender; Model 2: additionally adjusted for Model 1 + marital status, education status, family history of diabetes, current alcohol drinkers, physical activity, BMI; Model 3: additionally adjusted for Model 1 + Model 2 + having meals with family; OR odds ratio, aOR adjusted odds ratio, CI confidence interval; *p*-value: *** *p* < 0.001, ** *p* < 0.01, * *p* < 0.05.

**Table 4:** Comparison of dietary behaviors by high frequency between diabetes and non-diabetes participants.

Dietary Behavior		Case (*n* = 150)		Control (*n* = 150)		*p*-Value
Having family meals	*n* (%)	66	(44.0)	34	(23.0)	<0.001 **
Skipping breakfast		12	(8.0)	4	(2.7)	0.043 *
Eating out		5	(3.3)	13	(8.8)	0.048 *
Eating prepared foods		15	(10.0)	15	(10.1)	0.969
Having snacks		26	(17.3)	17	(11.4)	0.144
Removing visible fat		62	(43.4)	54	(37.5)	0.312
Drinking alcohol		1	(0.7)	0	(0.0)	0.323

Note: Chi-squared test was used to compare categorical variables; High frequency: doing every day; %: ratio of valid responses; *p*-value for the comparison between two groups: ** *p* < 0.01, * *p* < 0.05. All *p*-values are two-sided.

**Table 5:** The association between having meals with family and type 2 diabetes mellitus among the urban residents in Yangon, Myanmar, 2019.

		Association of Dietary Habits to T2DM		
	OR	aOR	95% CI	*p*-Value
**Having meals with family**				
Crude	2.63		1.60–4.35	<0.001 ***
Model 1		3.08	1.76–5.39	<0.001 ***
Model 2		2.50	1.37–4.56	0.003 **
Model 3		2.23	1.05–4.71	0.036 *

Note: Multivariable logistic regression analysis; Goodness-of-fit test for a logistic regression model 3; Pearson chi-squared test (*p* = 0.465), Hosmer–Lemeshow chi-squared test (*p* = 0.106); Crude: unadjusted; Model 1: adjusted for age, gender; Model 2: additionally adjusted for Model 1 + marital status, education status, family history of diabetes, current alcohol drinkers, physical activity, BMI; Model 3: additionally adjusted for Model 1 + Model 2 + seasonings + vegetables + fruit; OR odds ratio, aOR adjusted odds ratio, CI confidence interval; *p*-value: *** *p* < 0.001, ** *p* < 0.01, * *p* < 0.05.

## Data Availability

The datasets used and/or analyzed during the current study are available from the corresponding author upon reasonable request.
